# Cerebrospinal fluid findings in patients with neurological manifestations in post-COVID-19 syndrome

**DOI:** 10.1007/s00415-023-12092-4

**Published:** 2023-11-24

**Authors:** Fabian Boesl, Yasemin Goereci, Ameli Gerhard, Benno Bremer, Vanessa Raeder, Finja Schweitzer, Uta Hoppmann, Janina Behrens, Judith Bellmann-Strobl, Friedemann Paul, Brigitte Wildemann, Sven Jarius, Harald Prüss, Heinrich J. Audebert, Clemens Warnke, Christiana Franke

**Affiliations:** 1 Charité – Universitätsmedizin Berlin, Corporate member of Freie Universität Berlin and Humboldt-Universität zu Berlin, Department of Neurology and Experimental Neurology, Berlin, Germany; 2 Department of Neurology, Faculty of Medicine and University Hospital Cologne, University of Cologne, Cologne, Germany; 3 Experimental and Clinical Research Center, A Cooperation Between the Max Delbrück Center for Molecular Medicine in the Helmholtz Association and Charité Universitätsmedizin Berlin, Berlin, Germany; 4 Charité – Universitätsmedizin Berlin, Corporate Member of Freie Universität Berlin and Humboldt-Universität zu Berlin, Experimental and Clinical Research Center, Berlin, Germany; 5 Max Delbrück Center for Molecular Medicine in the Helmholtz Association (MDC), Berlin, Germany; 6 Molecular Neuroimmunology Group, Department of Neurology, University of Heidelberg, Heidelberg, Germany; 7 German Center for Neurodegenerative Diseases (DZNE), Berlin, Germany

**Keywords:** Post-COVID, Long-COVID, Cerebrospinal fluid, Blood–CSF barrier dysfunction, Neurology, Post-acute sequelae of SARS-CoV-2 infection

## Abstract

**Background:**

Information on cerebrospinal fluid (CSF) findings in patients with neurological manifestations in post-COVID-19 syndrome is scarce.

**Methods:**

Retrospective evaluation of 84 CSF samples in patients fulfilling post-COVID-19 criteria in two neurological post-COVID-19 outpatient clinics.

**Results:**

In 68% of samples, all CSF parameters were normal. The most frequent pathological CSF finding was elevation of total protein (median total protein 33.3 mg/dl [total range 18.5–116.2]) in 20 of 83 (24%) samples. The second most prevalent pathological finding was a blood–CSF barrier dysfunction as measured by elevation of QAlb (median QAlb 4.65 [2.4–13.2]) in 11/84 (13%). Pleocytosis was found in only 5/84 (6%) samples and was mild in all of them. CSF-restricted oligoclonal bands were found in 5/83 (6%) samples. Anti-neuronal autoantibodies in CSF were negative in most cases, whilst 12/68 (18%) samples were positive for anti-myelin autoantibodies in serum. PCR for herpesviridae (HSV-1/-2, VZV, EBV, CMV, HHV6) showed, if at all, only weakly positive results in CSF or EDTA whole blood/plasma.

**Conclusions:**

The majority of samples did not show any pathologies. The most frequent findings were elevation of total protein and blood–CSF barrier dysfunction with no signs of intrathecal inflammation. CSF analysis still keeps its value for exclusion of differential diagnoses.

## Background

After acute infection with severe acute respiratory syndrome coronavirus 2 (SARS-CoV-2), about 10–20% of patients report persisting or new-onset, often polytope, symptoms that are summarised as post-COVID-19 syndrome (PCS) [21]. PCS is defined as the continuation or development of new symptoms three months after a SARS-CoV-2 infection, with these symptoms lasting for at least two months with no other explanation [21].

A large proportion of PCS-associated symptoms can be attributed to the field of neurology or neuropsychiatry [4, 5]. Frequently reported neurological and neuropsychiatric PCS symptoms are cognitive impairment, fatigue, headaches, myalgia, sensory dysfunction and persisting loss of taste and smell [2]. Several patho-mechanisms including neuro-inflammation, autoimmunity, reactivation of neurotropic viruses, endothelial dysfunction and coagulopathy, neuro-invasion and metabolic dysfunctions have been suggested as potential causes of PCS [13]. To date, the exact aetiopathogenesis of PCS is still to be determined. Analysis of cerebrospinal fluid (CSF) is a standard diagnostic tool in neurological diseases and is part of the diagnostic work-up of persisting cognitive deficits. Whilst comprehensive data on CSF findings in patients with neurological manifestations during acute COVID-19 have previously been published, equivalent data for patients with neurological symptoms in PCS are missing so far [10]. Here, we present a systematic evaluation of 84 CSF analyses in PCS patients with neurological/neuropsychiatric symptoms.

## Methods

### Patients

Lumbar punctures (LPs) were performed in patients presenting in the neurological outpatient clinics of the German university hospitals in Cologne and Berlin as part of a clinical routine work-up. Results, patients’ characteristics and symptoms were analysed retrospectively from patient records. Only patients fulfilling the WHO consensus criteria for PCS and with PCR proof of prior SARS-CoV-2 infection were included in the study. Patients with other pre- or co-existing acute or chronic neurological, psychiatric or systemic autoimmune disorders were excluded. LPs were performed between 10/2020 and 05/2023. All participants gave informed written consent for the diagnostic procedure. This study was approved by the ethics committee of Charité – Universitätsmedizin Berlin (EA2/066/20 and EA2/102/22) and the ethics committee of the University of Cologne (20–1501).

## Methods

Methods are in compliance with existing German guidelines on CSF diagnostics and previously reported studies on CSF findings in acute COVID-19 [10, 14, 25, 26].

### Evaluation of blood–CSF barrier function

The blood-CSF-barrier (BCB) function was evaluated via the CSF/serum albumin quotient (QAlb = Alb_CSF_[mg/l]/Alb_serum_[g/l]. Albumin was assessed by immuno-nephelometry. The age-dependent upper reference limit of QAlb, *Q*_lim_(Alb), was calculated with following formula: *Q*_lim_(Alb) = 4 + (age/15). QAlb > Q_lim_(Alb) was interpreted as BCB dysfunction [16–18].

### Evaluation of humoral immune response

Isoelectric focussing was performed to determine oligoclonal immunoglobin G (IgG) bands (OCB) and evaluation followed international consensus [1]. Immunoglobulins were assessed by immunonephelometry. To quantitatively assess the degree of intrathecal immunoglobulin synthesis, the CSF/serum quotients for IgG (QIgG), IgM (QIgM), and IgA (QIgA) were calculated, with QIg = Ig_CSF_[mg/l]/Ig_serum_[g/l].

Reiber’s revised hyperbolic function was used to calculate *Q*_lim_(IgG), *Q*_lim_(IgM), and *Q*_lim_(IgA), the upper limits of the respective reference ranges, against QAlb [15]. Intrathecal immunoglobulin synthesis was assumed, if values for QIg surpassed *Q*_lim_(Ig) [15]. The absolute amount of locally, i.e. intrathecally, produced immunoglobulins (Ig-loc) and their fraction (in %; Ig-IF) were determined with the following equations: Ig-loc[mg/L] = [QIg − *Q*_lim_(Ig)] × Ig_serum_ and Ig-IF[%] = Ig-loc/Ig_CSF_ × 100 = [1 – *Q*_lim_(Ig)/QIg] × 100 [15].

### Cytological examination, CSF total protein and CSF l-lactate

CSF pleocytosis or increased CSF white cell count (WCC) was determined as ≥ 5/µl [14, 25, 26]. The age-dependent upper reference limit for CSF L-lactate was set at 2.1 mmol/l (16–50 years) and 2.6 mmol/l (> 50 years) [14, 25, 26]. For CSF total protein (TP), an upper reference level of 0.45 mg/l was used [14, 25, 26].

### Other markers

Autoantibodies against intracellular and surface antigens relevant for central nervous system diseases included antibodies against amphiphysin, collapsin response mediator-protein 5 (CRMP5), glutamic acid decarboxylase 65 (GAD65), Hu, paraneoplastic antigen Ma2/Ta (PNMA2), Ri, Tr/Delta and Notch-like epidermal growth factor-related receptor (DNER), Yo, α-amino-3-hydroxy-5-methyl-4-isoxazolepropionic acid receptor 1/2 (AMPA1/2-R), dipeptidyl-peptidase-like protein-6 (DPP6), G protein-coupled receptors B for gamma-aminobutyric acid (GABA-B-R), glycine receptor, Leucine-rich, glioma inactivated protein 1 (LGI1), myelin, metabotropic glutamate receptor 5 (mGluR5), contactin-associated protein-like 2 (CASPR2), dopamine-2 receptor, aquaporin-4 (AQP 4) and *N*-methyl-d-aspartate receptor (NMDAR) and were assessed in CSF and serum by immunohistochemistry (IHC), immunoblots, ELISA and cell-based assays. Qualitative PCR for DNA for herpesvirus (HSV) 1/2, varicella zoster virus (VZV) and human herpes virus 6 (HHV6) were performed in patients’ CSF and plasma. Quantitative PCR was performed for cytomegalovirus (CMV) and Epstein–Barr virus (EBV) in patients’ CSF and plasma (CMV) or whole blood (EBV).

### Quotient diagrams

QIgG, QIgA and QIgM were each plotted against QAlb as so-called quotient diagrams using the CSF Research Tool v3.0 (CoMed GmbH, Soest, Germany) [15, 18, 25]. As QAlb reference values are age-dependent, quotient diagrams are usually used to demonstrate BCB disruption and intrathecal Ig synthesis for one patient or patient groups of the same age. The upper reference limit for QAlb in the plots was calculated with the mean age of our cohort (47 years).

### Statistics

Samples were analysed in total, i.e. no subgroup analyses were performed. Spearman’s rho was used to assess correlations. *p* values < 0.05 were considered statistically significant.

## Results

A total of 84 lumbar punctures in 84 patients, including 53 females (63%), were evaluated for this study (72 Berlin, 12 Cologne). Mean age was 46.7 years (range 19–70 years). Most patients had a mild acute COVID-19 course, defined as no need of hospitalisation or oxygen supplementation during acute infection (*n* = 71; 84%). The mean time between positive SARS-CoV-2 PCR and LP was 290 days (range 100–831 days). The most frequent symptoms leading to LP were self-reported cognitive deficits (*n* = 75; 89%) and/or persisting fatigue (*n* = 63: 75%). Patients’ demography and reported symptoms are summarised in Table 1.

**Table 1:** Patients’ demographic and symptoms

Number of patients	84
*Age (years), median (range)*	46.5 (19–70)
*Gender*	
Female	53 (63%)
Male	31 (37%)
Non-binary	0
*Course COVID-19*	
Mild	71 (84%)
Moderate	3 (4%)
Severe	10 (12%)
*Time between COVID-19 and spinal tap (days)*	290 (100–831)
*Symptoms*	
Cognitive deficits	75 (89%)
Fatigue	63 (75%)
Headache	25 (30%)
Myalgia	22 (26%)
Pain syndromes	22 (26%)
Sleep disorder	21 (25%)
Sensory dysfunction	18 (21%)
Vertigo	16 (19%)
Loss of smell/taste	15 (18%)

**Fig. 1:**
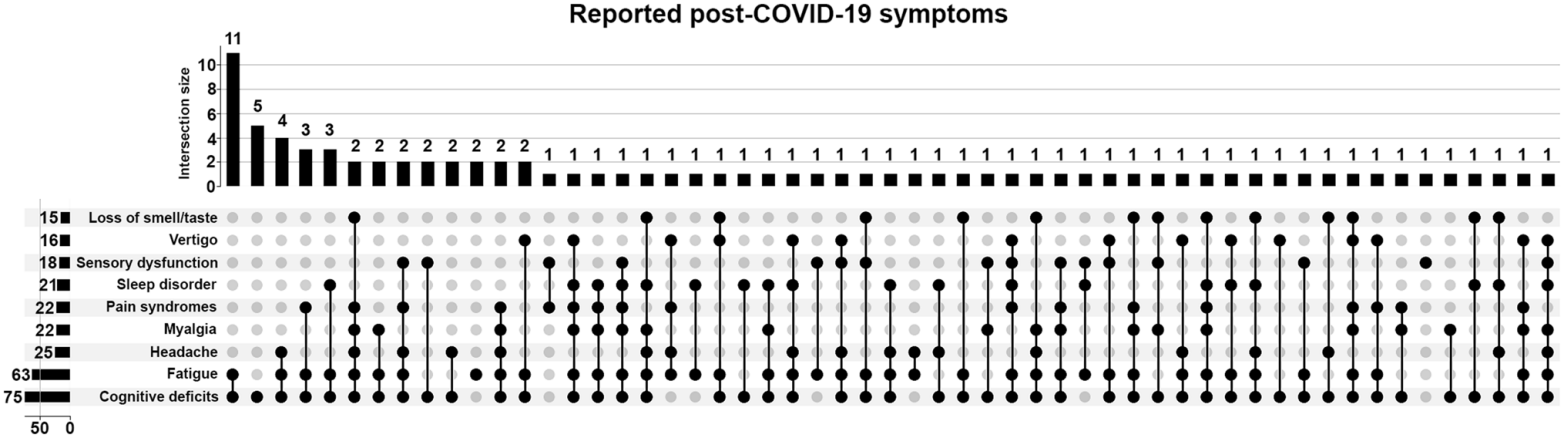
Post-COVID-19 symptoms plotted as an upset plot to show intersections

Symptoms were additionally plotted as an upset plot in Fig. 1 to show symptom intersections [12]. Absolute sample numbers differ for some of the sub-analyses since not all parameters were available in all patients, due to the retrospective nature of this study. The majority of patients had no pathological results in CSF analysis. Regarding WCC, CSF TP, CSF l-lactate, QAlb and QIgG/A/M, 54 of those 80 (68%) samples in which all of these parameters were assessed had entirely normal values.

### Blood–CSF barrier function

Elevation of QAlb, a sign of disruption of the BCB, was found in 11/84 (13%) of patients. In patients with BCB disruption, median QAlb was 8.2 (range: 6.8–13.2) (Table 2). No significant correlation between QAlb and duration of symptoms since COVID-19 onset was found (*r*  = − 0.62, *p* = 0.573, *n* = 84).

**Table 2:** Blood–CSF barrier function, CSF and serum albumin, CSF total protein and CSF l-lactate

*Blood–CSF barrier function*		
QAlb > *Q*_lim_(Alb)		11/84 (13%)
QAlb, all LPs		4.65 (2.4–13.2; 84)
QAlb, if positive		8.2 (6.8–13.2; 11)
Alb CSF	mg/l	218 (101–573; 84)
Alb serum	g/l	46.6 (40–68; 84)
Albuminocytological dissociation		10/83 (12%)
Combined intrathecal synthesis and BCB disruption		0/81 (0%)
*CSF total protein*		
CSF TP, elevated		20/83 (24%)
CSF TP, all LPs	mg/dl	33.3 (18.5–116.2; 83)
CSF TP, if elevated	mg/dl	54.75 (46.5–116.2; 20)
CSF TP, > 100 mg/dl		1/83 (1%)
*CSF l-lactate*		
CSF l-lactate, elevated		1/83 (1%)
CSF l-lactate, all LPs	mmol/l	1.59 (1.2–2.7; 83)
CSF l-lactate, if elevated	mmol/l	2.7 (2.7–2.7; 1)

Results are given as medians (with ranges and sample or patient numbers in brackets) and frequencies (with percentages in brackets), respectively

*Alb* albumin, *BCB* blood–CSF barrier, *LP* lumbar puncture, *QAlb* CSF/serum albumin ratio, *TP* total protein

### CSF total protein

**Fig. 2:**
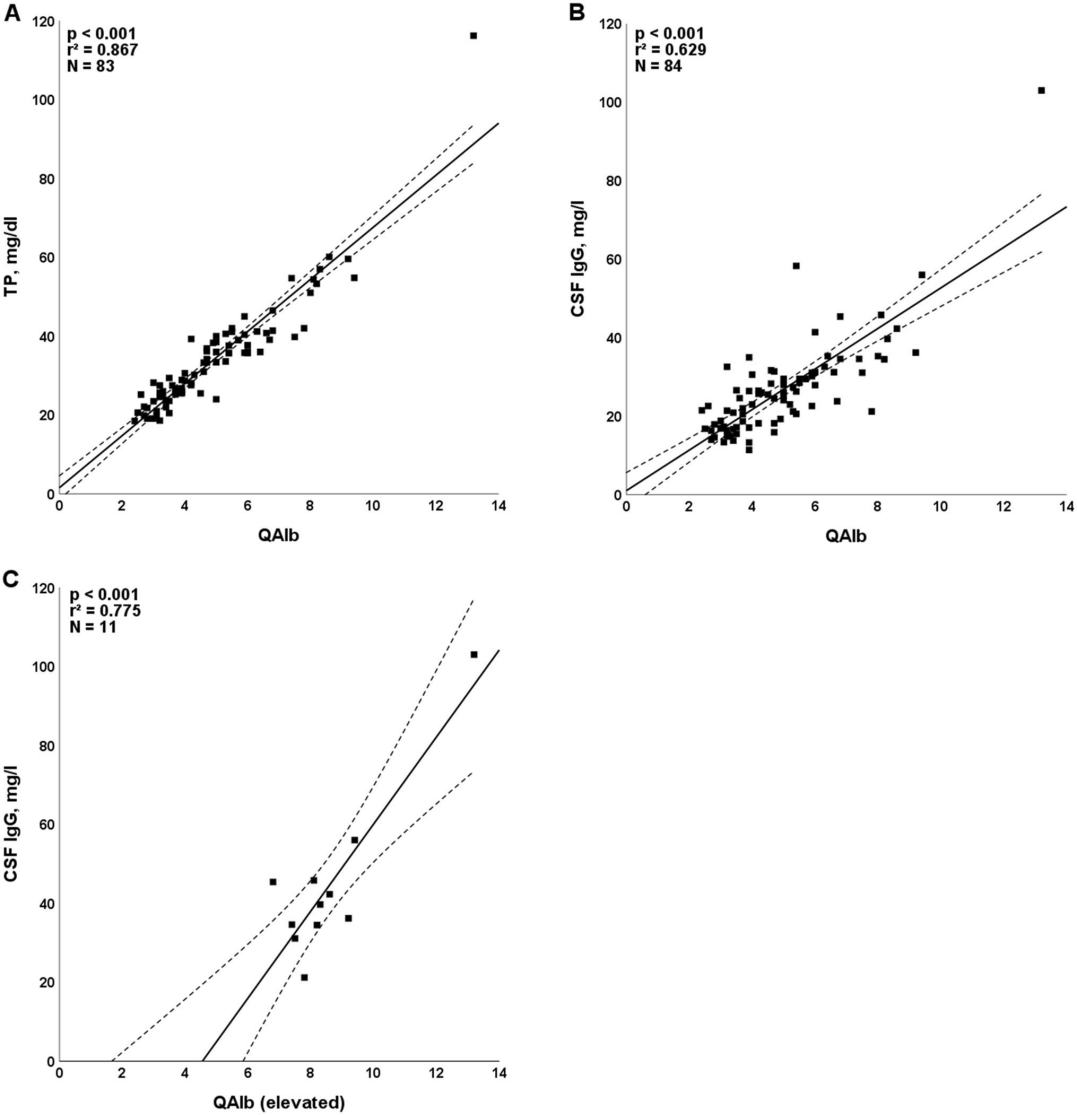
Regression analyses of CSF total protein (**A**) and CSF IgG concentrations (**B, C**), respectively, and QAlb, demonstrating a close relationship between these parameters and QAlb. Solid lines indicate medians. Dotted lines represent the upper and lower 95% confidence bands of the regression line. *IgG* immunoglobulin G, *N* number of samples, *QAlb* albumin CSF/serum ratio, *TP* total protein

Elevated concentrations of CSF TP were detected in 20/83 (24%) patients (median 54.75 mg/dl; range 46.5–116.2 mg/dl) (Table 2). CSF TP values were highly dependent on QAlb in linear regression analysis (*r* = 0.931, *r*^2^ = 0.867, *p* < 0.001, *n* = 83) (Fig. 2A). Elevated TP values were > 45 und < 50 mg/dl (“borderline”) in 1/83 (1%) samples, ≥ 50 and ≤ 100 in 8/83 (10%) samples and > 100 mg/dl in 1/83 (1%) samples. As QAlb, TP did not significantly correlate with time since COVID-19 onset (*r* = − 0.64, *p* = 0.568, *n* = 83).

### Cellular immune response

Pleocytosis was only detected in 5/84 (6%) patients with a median of 7 cells/µl (Table 3). Only one patient exhibited a WCC > 10 cells/µl (16 cells/µl). No relevant blood contamination was found in samples with pleocytosis. Lymphocytes were the predominant cell type in patients with pleocytosis. None of the patients with pleocytosis exhibited a BCB dysfunction according to QAlb. Three of the patients with pleocytosis had otherwise normal CSF results. One patient exhibited intrathecal IgG synthesis > 10% and positive OCB, another intrathecal IgA synthesis, albeit < 10%.

**Table 3:** CSF white cell counts and cytology

Pleocytosis	Samples	5/84 (6%)
WCC, all samples	Cells/µl	2 (0–16; 84)
WCC, if elevated	Cells/µl	7 (5–16; 5)
WCC, ≥ 100	Samples	0/84 (0%)
Lymphocytes	Samples	69/69 (100%)
Monocytes	Samples	69/69 (100%)
Neutrophils	Samples	7/69 (10%)
Eosinophils	Samples	0/69 (0%)
Basophils	Samples	0/69 (0%)
Plasma cells	Samples	0/69 (0%)
Lymphoid cells	Samples	0/69 (0%)
Macrophages	Samples	1/69 (1%)
No pleocytosis	Samples	79/84 (94%)

Results are given as medians (with ranges and sample or patient numbers in brackets) and frequencies (with percentages in brackets), respectively

*WCC* white cell count

### Albuminocytological dissociation

All of the 20 patients with elevated CSF TP levels had a normal CSF WCC (so-called albuminocytological dissociation [ACD]), corresponding to 24% of all patients with available data. Interpreting ACD as elevated QAlb without pleocytosis, 11/84 (13%) patients showed ACD.

### CSF l-lactate

Only one patient had slightly increased levels of CSF L-lactate with 2.7 mmol/l. With a WCC of 1 cell/µl, neutrophil pleocytosis could be ruled out as cause of CSF L-lactate elevation. The patient also had elevated CSF glucose levels (6.8 mmol/l) and CSF TP elevation (51 mg/dl) without BCB disruption. In contrast to CSF TP values, CSF L-lactate levels did not show a statistically significant relation to QAlb in regression analysis (*r* = 0.188, *r*^2^ = 0.035, *p* = 0.089, *n* = 83).

### Intrathecal IgG synthesis

CSF-restricted OCB were found in 5/83 (6%) patients (pattern 2 in 4/83 [5%], pattern 3 in 1/83 [1%]), consistent with intrathecal IgG synthesis (Table 4) [1, 25, 29]. Identical OCB in serum and CSF with no CSF-restricted bands (pattern 4) were found in 4/83 patients (5%). Another though less sensitive marker for intrathecal IgG synthesis, QIgG, was elevated in 5/84 (6%). In those five cases, only one patient had CSF-restricted OCB (type 2). This patient is also the only case where the intrathecal IgG fraction surpassed 10% (10.36%). Levels below 10% are not reliably demonstrating intrathecal synthesis according to current guidelines due to imprecision of nephelometric IgG testing [14]. CSF IgG concentrations surpassed the upper reference limit of 40 mg/l in 7/84 (8%) patients. Only one of these patients had positive OCB and elevated QIgG. In the other cases, QIgG was normal, signifying that elevated CSF IgG concentrations were not caused by intrathecal synthesis, but by passive transfer of IgG from the periphery to the CSF. This is corroborated by elevated QAlb as a sign of BCB dysfunction in 5 of the 6 (83%) remaining cases. In general, QAlb was able to predict CSF IgG significantly in regression analysis in patients with elevated QAlb (*r* = 0.881, *r*^2^ = 0.775, *p* < 0.001, *n* = 11) as well as in the total cohort (*r* = 0.793, *r*^2^ = 0.629, *p* < 0.001, *n* = 84) (Fig. 2B, C).

**Table 4:** Frequency of intrathecal IgG synthesis, oligoclonal IgG patterns, IgG CSF/serum ratios, intrathecal IgG fractions, absolute amount of locally produced IgG, and absolute IgG concentrations in the CSF and serum

	Units	Total
*Intrathecal IgG synthesis*		
OCB positive or IgG-IF ≥ 10%	Samples	5/83 (6%)
OCB positive	Samples	5/83 (6%)
OCB pattern 1	Samples	74/83 (89%)
OCB pattern 2	Samples	4/83 (5%)
OCB pattern 3	Samples	1/83 (1%)
OCB pattern 4	Samples	4/83 (5%)
OCB pattern 5	Samples	0/83 (0%)
QIgG > *Q*_lim_(IgG)	Samples	5/84 (6%)
QIgG, all LPs	–	2.25 (1.2–7.5; 84)
QIgG, if positive	–	2.80 (1.7–4.3; 5)
IgG-IF, all LPs	% IgG(CSF)	0 (0–10.36; 84)
IgG-IF, QIgG positives	% IgG(CSF)	4.52 (0–10.36; 5)
IgG-IF, > 10%	Samples	1/84 (1%)
IgG-loc, all LPs	mg/l	0 (0–6.04; 84)
IgG-loc, QIgG positives	mg/l	1.35 (0–6.04; 5)
IgG CSF, all LPs	mg/l	24.95 (11.4–103; 84)
IgG CSF, QIgG positives	mg/l	30.60 (16.7–58.3; 5)
IgG serum, all LPs	g/l	11 (5.8–16.5; 84)
IgG serum, QIgG positives	g/l	13.1 (9.76–15.2; 5)
IgG serum, elevated	Samples	0/84 (0%)
IgG index positive	Samples	6/84 (7%)
IgG index	Index	0.44 (0.41–0.47; 84)

Quotients, indices, concentrations and fractions are given as medians (with ranges and sample numbers in brackets)

*OCB* oligoclonal IgG bands, *QIgG/A/M* CSF/serum IgG/A/M ratio, *IgG/A/M-IF* intrathecally produced IgG/IgA/IgM fraction; *IgG/A/M-loc* locally (intrathecally) produced IgG/A/M; *LP* lumbar puncture

### Intrathecal IgA synthesis

QIgA was elevated in only 1/81 (1%) patients with available data (Table 5). This patient had a mild pleocytosis of 8 cells/µl. Yet, the intrathecal IgA fraction (7.9%) and the absolute amount of intrathecally produced IgA (0.17 mg/l) were rather low. Elevation of serum IgA levels (> 4 g/l) was noted in 4/81 (5%) samples.

**Table 5:** Immunoglobulin class response patterns (ICRPs)

	Units	Total
*a. Based on QIg > Q*_*lim*_*(Ig)*		
3-class reaction	Samples	0/81 (0%)
2-class reaction	Samples	0/81 (0%)
IgG + IgM	Samples	0/81 (0%)
IgG + IgA	Samples	0/81 (0%)
IgM + IgA	Samples	0/81 (0%)
1-class reaction	Samples	6/81 (7%)
Only IgG	Samples	4/81 (5%)
Only IgM	Samples	1/81 (1%)
Only IgA	Samples	1/81 (1%)
*b. Based on Ig-IF > 10%*		
3-class reaction	Samples	0/81 (0%)
2-class reaction	Samples	0/81 (0%)
IgG + IgM	Samples	0/81 (0%)
IgG + IgA	Samples	0/81 (0%)
IgM + IgA	Samples	0/81 (0%)
1-class reaction	Samples	2/81 (2%)
Only IgG	Samples	1/81 (1%)
Only IgM	Samples	1/81 (1%)
Only IgA	Samples	0/81 (0%)

### Intrathecal IgM synthesis

CSF IgM was below detection limit in 30 cases, but QIgM was < *Q*_lim_(IgM) even if applying the lowest CSF IgM level detectable nephelometrically. QIgM was elevated in just 1/82 patients (Table 5). This patient had an intrathecal IgM fraction of 30.9%, surpassing the threshold of 10% and an absolute amount of intrathecally produced IgM of 0.19 mg/l. No erythrocytes were present in this sample, so an effect of blood contamination on QIgM can be ruled out [14]. Serum IgM levels were elevated in only 2/82 (2%) samples.

### Immunoglobulin (Ig) class patterns

**Fig. 3:**
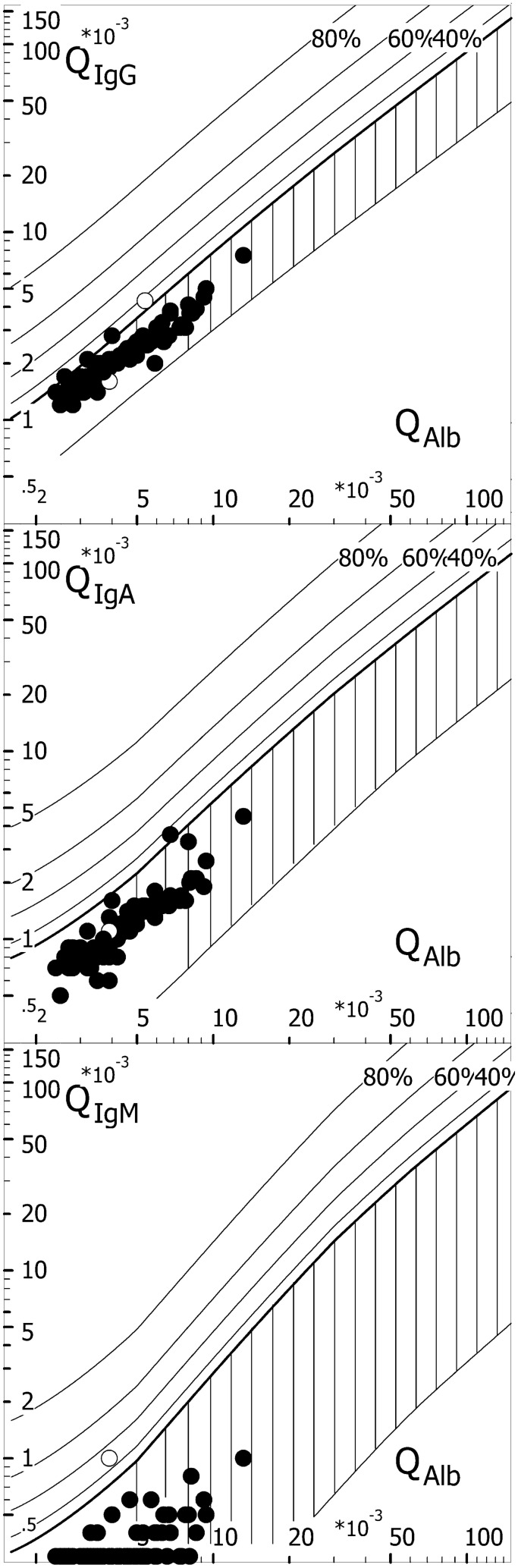
CSF/serum quotient diagrams for IgG, IgM and IgA. Individual CSF/serum ratios of IgG, IgA and IgM are plotted against CSF/serum albumin ratios. Values above the upper hyperbolic discrimination line, *Q*_lim_, indicate intrathecal synthesis of the respective immunoglobulin (Ig) class. Individual intrathecal fractions, Ig-IF, can be directly read by interpolation from the percentiles above *Q*_lim_ (median values are given in Tables 4 and 5). Open circles represent samples with Ig-IF values > 10%. Graphs were created using CSF Research Tool v3.0 (CoMed GmbH, Soest, Germany). *IgG/A/M* immunoglobulin G/A/M, *QIgG/A/M* CSF/serum IgG/A/M ratios, *QAlb* CSF/serum albumin ratio

None of the 81 patients displayed a three-class reaction, which is defined as elevation of QIgG, QIgA and QIgM, or a two-class reaction, which is defined by elevation of two of these three parameters, based on QIg > *Q*_lim_(Ig) (Table 5). Based on Ig CSF/serum ratios, intrathecal Ig synthesis was restricted to one immunoglobulin class in 6/81 samples (7%). Based on Ig-IF > 10%, intrathecal Ig synthesis was restricted to one immunoglobulin class in 2/81 samples (2%). QIgG, QIgA and QIgM were each plotted against QAlb as a quotient diagram to visualise the lack of intrathecal immunoglobulin synthesis in almost all cases (Fig. 3).

### CSF PCR for herpesviridae

Reactivation of latent herpesviridae is one of the hypothesised pathomechanisms in PCS [13]. CSF and EDTA whole blood or plasma samples were tested for HSV-1/-2, VZV, EBV, CMV and HHV6 (Table 6). Only one weakly positive PCR result of HSV-2 was detected in CSF with the detection limit of the PCR test being 400 copies per millilitre. This patient did not exhibit clinical signs of acute HSV encephalitis and had a normal WCC and CSF l-lactate level, arguing against acute encephalitis. Furthermore, no BCB dysfunction was observed, but OCB were positive (pattern 2). EDTA whole blood/plasma PCR results in this patient were all negative. In EDTA whole blood/plasma, but not in the CSF, six further patients had a weakly positive herpesviridae PCR result (4 × EBV with less than 1000 copies per millilitre, 1 × HSV1, 1 × HHV6).

**Table 6:** CSF and EDTA whole blood/plasma PCR results for herpes simplex virus (HSV) 1 and 2, varicella zoster virus (VZV), Epstein–Barr virus (EBV), cytomegalovirus (CMV), human herpes virus 6 (HHV6)

	Positive CSF PCR	Positive EDTA PCR
HSV-1	0/69 (0%)	1/55 (2%)*
HSV-2	1/69 (1%)*	0/54 (0%)
VZV	0/67 (0%)	0/54 (0%)
EBV	0/69 (0%)	4/55 (7%)*
CMV	0/69 (0%)	0/55 (0%)
HHV6	0/65 (0%)	1/55 (2%)*
Sum	1/408	6/328

*Weakly positive PCR result

### Anti-neuronal autoantibodies

CSF and/or serum samples were tested for anti-neuronal autoantibodies using commercial assays. Myelin autoantibodies could be detected by IHC in 12/68 tested serum samples (18%) with titers ranging from 1:100 (*n* = 11) to 1:1000 (*n* = 1) (Table 7). Other positive results for anti-neural autoantibodies could only be observed in individual patients and could all not be confirmed by other assays: one patient with a positive immunoblot for GAD-65 antibodies in CSF and serum, and one with GAD65 antibodies in serum, who also had an anti-myelin titre of 1:100 in serum. However, these positive GAD65 immunoblot results could not be confirmed by IHC. Two other patients had positive serum immunoblot results for either Ma2/Ta autoantibodies or Yo-autoantibodies, which also could not be confirmed by IHC. Another female patient had a strongly positive serum Yo-autoantibody immunoblot result, which could initially be confirmed by IHC. However, in follow-up measurement, IHC was negative and tumour screening was negative. None of these 16 patients had pleocytosis, rather arguing against acute autoimmune encephalitis. Two patients with anti-myelin antibodies exhibited BCB dysfunction according to QAlb (QAlb = 7.5 with *Q*_lim_(Alb) = 6.93; QAlb = 9.2 with *Q*_lim_(Alb) = 7.47). None of the 16 patients demonstrated intrathecally Ig synthesis according to IgIF and QIgG/A/M.

**Table 7:** Anti-neuronal and anti-glial autoantibody findings

	CSF, positive	Serum, positive
Amphiphysin-IgG	0/70 (0%)	0/71 (0%)
CV2/CRMP5-IgG	0/70 (0%)	0/71 (0%)
GAD65-IgG	1/67 (2%)*	2/68 (3%)*
Hu-IgG	0/70 (0%)	0/71 (0%)
Ma2/Ta (PNMA2)-IgG	0/70 (0%)	1/71 (1%)*
Ri-IgG	0/70 (0%)	0/71 (0%)
Tr/DNER-IgG	0/67 (0%)	0/68 (0%)
Yo-IgG	0/70 (0%)	2/71 (2%)^#^
AMPA1-R (GluA1)-IgG	0/73 (0%)	0/73 (0%)
AMPA2-R (GluA2)-IgG	0/73 (0%)	0/73 (0%)
DPP6 IgG	0/68 (0%)	0/69 (0%)
GABA-B-R-IgG	0/73 (0%)	0/73 (0%)
Glycine-R-IgG	0/67 (0%)	0/68 (0%)
LGI1-IgG	0/73 (0%)	0/73 (0%)
Myelin-IgG	0/67 (0%)	12/68 (18%)
mGluR5-IgG	0/67 (0%)	0/68 (0%)
CASPR2-IgG	0/73 (0%)	0/73 (0%)
Dopamine-2-R-IgG	0/67 (0%)	0/68 (0%)
AQP4-IgG	0/69 (0%)	0/70 (0%)
NMDAR-IgG	0/73 (0%)	0/73 (0%)
Sum	1/1397 (0%)	17/1411 (1%)

*CRMP5* Collapsin response mediator-protein 5, *GAD65* glutamic acid decarboxylase 65, *PNMA2* paraneoplastic antigen Ma2, *DNER* Delta and Notch-like epidermal growth factor-related receptor, *AMPA1/2-R* α-amino-3-hydroxy-5-methyl-4-isoxazolepropionic acid receptor, *DPP6* dipeptidyl-peptidase-like protein-6, *GABA-B-R* G protein-coupled receptors B for gamma-aminobutyric acid, *LGI1* Leucine-rich, glioma inactivated protein 1, *mGluR5* metabotropic glutamate receptor 5, *CASPR2* contactin-associated protein-like 2, *AQP4* aquaporin-4, *NMDAR* *N*-methyl-d-aspartate receptor

*Positive results in immunoblot were not confirmed via immunohistochemistry (IHC)

^#^One of the positive Yo-IgG results in immunoblot was not confirmed via IHC

## Discussion

Underlying patho-mechanisms of PCS and neurological symptoms in PCS in particular are yet to be determined. To our knowledge, this study is so far the most comprehensive analysis of CSF findings in patients with neurological symptoms in PCS.

Most patients did not show pathologies in CSF analysis and thus a distinct CSF phenotype for PCS could not be established in our cohort. Neurological manifestations in PCS seem not to be associated with persistent central nervous inflammation and quantitative or qualitative immunoglobulin synthesis in the vast majority of cases. Since median time between SARS-CoV-2 PCR and spinal tap was 290 days, a previous intrathecal inflammation in the acute phase of COVID-19 or at an earlier stage of PCS cannot be ruled out. However, a previous study of CSF findings in patients with acute COVID-19 and neurological symptoms did not find evidence of intrathecal inflammation in most cases as well [10].

The most frequent finding in our cohort was elevation of TP and BCB dysfunction. Whilst the elevation of TP in our patients can be explained by the BCB dysfunction, as demonstrated by a highly significant correlation of CSF TP with QAlb, the reason for the BCB dysfunction in our cohort remains unclear. We hypothesise that these BCB dysfunctions might be related to PCS. BCB dysfunction was also prevalent, albeit with a much higher frequency, in a large cohort of patients with acute COVID-19 [10]. In that study, QAlb was elevated in 58/116 (50%) samples and remained elevated > 14 days (48%) and even > 30 days (56%) after onset of neurological symptoms. BCB disruption may also occur in the absence of intrathecal inflammation. In non-inflammatory neurological diseases, the frequency of isolated BCB dysfunction was 18% in a large European cohort [3]. In acute COVID-19, there are several presumed mechanisms that may lead to BCB dysfunction, such as direct interactions of SARS-CoV-2 with brain endothelial cells, hypoxia, COVID-19-associated coagulopathy, and a severe systemic inflammatory response due to elevation of pro-inflammatory cytokines, chemokines and acute-phase proteins due to SARS-CoV-2 infection [6]. In PCS patients, evaluation of intracellular cytokine production after stimulation demonstrated significant elevation in the production of IL-2 and IL-6 amongst CD4^+^ and CD8^+^ T cells, and of IL-4 from CD4^+^ T cells compared to matched control groups [11]. Microcerebrovascular endothelial cells, which contribute to BCB function, express receptors for proinflammatory cytokines, including IL-6 [8, 27]. In acute COVID-19, a significant increase in serum and CSF levels of IL-6 and other cytokines could be demonstrated, which was often associated with BCB dysfunction, partly remaining positive at high levels for weeks and, in the few patients with long-term data tested, even months [10]. A delayed restoration of BCB functioning after disruption due to COVID-19 or even ongoing dysfunction due to elevated proinflammatory cytokines could thus be an explanation for neurological manifestations in PCS in some patients. As no LP was performed in our patients before the onset of PCS, it is unknown whether BCB dysfunction was present already during acute COVID-19 or developed later. Moreover, the time between onset of COVID-19 and CSF analysis was rather long in our cohort. Further studies are needed that investigate the dynamics of QAlb over the course of PCS.

Given that QAlb was elevated only in a subset of patients, other patho-mechanisms should still be considered. Persistence of SARS-CoV-2 RNA has been hypothesised as a cause of PCS symptoms. Analysis of SARS-CoV-2-specific antibody index and SARS-CoV-2 RNA in CSF in PCS patients did not show signs of a persistent infection of the central nervous system [20]. Similarly, SARS-CoV-2-PCR in CSF was also negative in 76/76 patient with acute COVID-19 in a recent study [10].

Another hypothesised aetiology of PCS is the reactivation of latent herpesviridae [13]. A previous study reported a significantly higher percentage of EBV reactivation in PCS patients, as based on positive serum titers for EBV early antigen-diffuse IgG or EBV viral capsid antigen IgM, compared to control groups in one PCS cohort [9]. Moreover, EBV viraemia during acute infection has been proposed as a predictor for the development of PCS symptoms [23]. However, it should be noted that other viral infections can cause reactivation of EBV [19], but disease patterns similar to PCS do not occur commonly in the aftermath. In our study, PCR testing for HSV-1/-2, VZV, EBV, CMV and HHV6 did not show evidence of manifest replication in the central nervous system (CNS) in almost all patients, rendering it unlikely that herpesvirus reactivation in the CNS played a major role in the pathogenesis of PCS in our patients. However, more in-depth serological analysis and evaluation of virus specific T cells are needed to better understand the role of herpesviridae in PCS.

Autoimmunity is another patho-mechanism discussed to underlie PCS. In our analysis of anti-neuronal autoantibodies against known antigens in patients’ CSF and serum, the most frequent finding were myelin autoantibodies in patients’ serum. High titres of myelin autoantibodies have also been found in healthy cohorts and their diagnostic value is controversial, leaving uncertainty if this finding really is involved in the aetiopathology of PCS [22]. The other positive autoantibody findings in CSF and serum, as determined by means of a commercial immunoblot, could not be confirmed by other methods, leaving the possibility of false-positive results. In another study, some of us could demonstrate anti-neuronal autoantibodies against undetermined antigens in more than half of the examined patients with persisting cognitive deficits after COVID-19 by use of an indirect immunofluorescence IHC assay employing rodent brain tissue sections [7]. Interestingly, the presence of autoantibodies in CSF correlated with the results of neurocognitive testing [7]. Autoantibodies against not yet determined central nervous antigens could thus be involved in the manifestation of neurological PCS symptoms. However, as a limitation, no control group was included and titers were not assessed in that study [7]. Moreover, the pathogenic relevance of these antibodies is still unknown and more studies addressing the role of autoimmunity in PCS are needed.

Though most of our samples did not exhibit any CSF pathologies, we still recommend including CSF analysis in the diagnostic work-up of patients with neurological symptoms and suspected PCS. Current definitions of PCS include that other diagnoses must be ruled out and CSF analysis is essential in neurological diagnostics [21]. Furthermore, the incidence of neurological and psychiatric diseases is elevated after COVID-19 when compared to influenza and other respiratory diseases, thus further arguing for an in-depth diagnostic work-up of reported neurological symptoms [24].

## Strength and limitations

The relatively high number of CSF samples in patients with neurological symptoms due to PCS according to the WHO Delphi consensus criteria is a strength of this study. Furthermore, patients with pre- or co-existing neurological, psychiatric and systemic autoimmune diseases were not included in this study to prevent inclusion of CSF alterations caused by other disorders. Although samples from two centres were included in this study, the majority of samples derived from Berlin, thus limiting the generalizability of our results. Furthermore, due to the retrospective nature of this study, not all parameters were assessed in all patients, leaving the possibility that not all results are representative for the total cohort. Another limitation is that due to the heterogeneity of reported symptoms and the low number of patients with moderate and severe COVID-19 courses, the formation of subgroups to evaluate statistical differences seemed unrewarding and was therefore not included. Comparison of CSF from PCS patients with and without neurological symptoms could provide further insight in understanding PCS but appears unethical due to the invasive nature of LP. Analysis of cytokines was not performed and should be the topic of further studies to help reveal further insights in the aetiopathogenesis of PCS. Additionally, not every potentially relevant CSF parameter was assessed in this study. For example, neurofilament light chain was not measured, although neurofilament light chain elevation in the acute phase of COVID-19 has been demonstrated [28], depicting it as a marker of interest for further evaluation in PCS. PCR testing for SARS-CoV-2 RNA was not performed in this study, since previously published data by our group could demonstrate that SARS-CoV-2 RNA was not detectable via PCR in the CSF of another cohort [20].

## Conclusions

In conclusion, our analysis shows that (1) elevation of TP is the most frequent pathological CSF finding in PCS with neurological manifestations, followed by (2) BCB dysfunction as indicated by elevated QAlb. (3) BCB dysfunction in PCS is generally not associated with intrathecal inflammation, as demonstrated by (4) the absence of pleocytosis, intrathecal immunoglobulin synthesis, elevated IgG, IgA, IgM ratios and CSF-restricted OCB in most patients. (5) Antineuronal autoantibodies against well-established autoantigens are rare in PCS, with the most frequent finding being serum anti-myelin autoantibodies with unclear diagnostic significance. (6) No proof of manifest replication of HSV-1/-2, VZV, EBV, CMV and HHV6 was found in patients’ CSF and blood by means of PCR analysis. (7) Most analyses did not show any pathological CSF findings, but (8) CSF analysis should be part of the diagnostic work-up of neurological symptoms in PCS to rule out possible differential diagnoses.

## Data Availability

The datasets used and/or analysed during the current study are available from the corresponding author on reasonable request.

## Abbreviations

ACD: Albuminocytological dissociation
AMPA1/2-R: α-Amino-3-hydroxy-5-methyl-4-isoxazolepropionic acid 1/2 receptor
AQP4: Aquaporin-4
BCB: Blood–CSF barrier
CMV: Cytomegalovirus
CASPR2: Contactin-associated protein-like 2
CRMP5: Collapsin response mediator protein 5
CNS: Central nervous system
CSF: Cerebrospinal fluid
D2: Dopamine receptor 2
DNER: Delta and Notch-like epidermal growth factor-related receptor
DPP6: Dipeptidyl-peptidase-like protein-6
EBV: Epstein–Barr virus
GABA-B-R: G protein-coupled receptors B for gamma-aminobutyric acid
GAD65: Glutamic acid decarboxylase 65
HHV6: Human herpes virus 6
HSV: Herpes simplex virus
IF: Intrathecal fraction
IHC: Immunohistochemistry
IgG/M/A: Immunoglobulin G/M/A
LGI1: Leucine-rich, glioma inactivated 1 protein
LP: Lumbar puncture
mGluR5: Metabotropic glutamate receptor 5
NMDAR: *N*-Methyl-d-aspartate receptor
OCB: Oligoclonal bands
QAlb: Albumin CSF/serum ratio
QIgG: IgG CSF/serum ratio
QIgM: IgM CSF/serum ratio
QIgA: IgA CSF/serum ratio
PCS: Post-COVID-19 syndrome
PNMA2: Paraneoplastic antigen Ma2
SARS-CoV-2: Severe acute respiratory syndrome coronavirus type 2
VZV: Varicella zoster virus
WCC: White cell count
